# Horizontal gene transfer in bdelloid rotifers is ancient, ongoing and more frequent in species from desiccating habitats

**DOI:** 10.1186/s12915-015-0202-9

**Published:** 2015-11-04

**Authors:** Isobel Eyres, Chiara Boschetti, Alastair Crisp, Thomas P. Smith, Diego Fontaneto, Alan Tunnacliffe, Timothy G. Barraclough

**Affiliations:** Department of Life Sciences, Imperial College London, Silwood Park Campus, Ascot, SL5 7PY UK; Department of Chemical Engineering and Biotechnology, University of Cambridge, New Museums Site, Pembroke Street, Cambridge, CB2 3RA UK; National Research Council, Institute of Ecosystem Study, Largo Tonolli 50, 28922 Verbania Pallanza, Italy; Department of Animal and Plant Sciences, University of Sheffield, Alfred Denny Building, Western Bank, Sheffield, S10 2TN UK

**Keywords:** Adaptation, Bdelloid rotifers, Evolution, Horizontal gene transfer, Lateral gene transfer, Metazoans

## Abstract

**Background:**

Although prevalent in prokaryotes, horizontal gene transfer (HGT) is rarer in multicellular eukaryotes. Bdelloid rotifers are microscopic animals that contain a higher proportion of horizontally transferred, non-metazoan genes in their genomes than typical of animals. It has been hypothesized that bdelloids incorporate foreign DNA when they repair their chromosomes following double-strand breaks caused by desiccation. HGT might thereby contribute to species divergence and adaptation, as in prokaryotes. If so, we expect that species should differ in their complement of foreign genes, rather than sharing the same set of foreign genes inherited from a common ancestor. Furthermore, there should be more foreign genes in species that desiccate more frequently. We tested these hypotheses by surveying HGT in four congeneric species of bdelloids from different habitats: two from permanent aquatic habitats and two from temporary aquatic habitats that desiccate regularly.

**Results:**

Transcriptomes of all four species contain many genes with a closer match to non-metazoan genes than to metazoan genes. Whole genome sequencing of one species confirmed the presence of these foreign genes in the genome. Nearly half of foreign genes are shared between all four species and an outgroup from another family, but many hundreds are unique to particular species, which indicates that HGT is ongoing. Using a dated phylogeny, we estimate an average of 12.8 gains versus 2.0 losses of foreign genes per million years. Consistent with the desiccation hypothesis, the level of HGT is higher in the species that experience regular desiccation events than those that do not. However, HGT still contributed hundreds of foreign genes to the species from permanently aquatic habitats. Foreign genes were mainly enzymes with various annotated functions that include catabolism of complex polysaccharides and stress responses. We found evidence of differential loss of ancestral foreign genes previously associated with desiccation protection in the two non-desiccating species.

**Conclusions:**

Nearly half of foreign genes were acquired before the divergence of bdelloid families over 60 Mya. Nonetheless, HGT is ongoing in bdelloids and has contributed to putative functional differences among species. Variation among our study species is consistent with the hypothesis that desiccating habitats promote HGT.

**Electronic supplementary material:**

The online version of this article (doi:10.1186/s12915-015-0202-9) contains supplementary material, which is available to authorized users.

## Background

Horizontal gene transfer (HGT), the “non-sexual movement of genetic material between two organisms” [1], is relatively common in prokaryotes [2] and single-celled eukaryotes, but a number of factors combine to make it far rarer in multicellular eukaryotes [3, 4]. In order for a eukaryotic species to gain a gene by HGT, foreign DNA must enter the host nucleus, integrate into the genome, and in more complex organisms it must enter the sequestered germline in order to be transmitted to offspring [5, 6]. Once there, it must not experience strong negative selection, despite potential for genetic incompatibility with the host genome and mismatch between the niche of the donor and the host [7]. Over the longer term, foreign DNA may become “domesticated” in the recipient genome and provide novel function [8].

Recent estimates indicate that, although occurring at a lower frequency than in prokaryotes, HGT in eukaryotes might be less rare than previously thought [9], and among metazoans there are now several well-documented cases of the horizontal transfer of single genes or small sets of genes from non-metazoan donors. For example, multiple horizontal transfer events are thought to have allowed the emergence of plant parasitism in root-knot nematodes, through the uptake of genes from bacteria used for plant cell wall degradation and modulation of plant defence [10, 11]. Several metazoans have also acquired the ability to synthesize carotenoids using genes of fungal origin [12, 13]. Despite these examples, the role of HGT in the generation of novel adaptations is often ignored when looking at the evolution of eukaryotes.

A recently discovered exception to the relatively low rate of HGT in multicellular eukaryotes was found in bdelloid rotifers. Bdelloids are microscopic, aquatic animals that have experienced extensive HGT. Since the initial discovery of inter-kingdom horizontally acquired genes in two species of bdelloid rotifer [14], work in *Adineta ricciae* has demonstrated that many of these genes are expressed, and 8–10 % of *A. ricciae* transcripts are potentially foreign [15]. These genes are involved in diverse metabolic pathways and can contribute to the desiccation response [16], which is important for bdelloids that live in ephemeral habitats such as the water film on terrestrial mosses. The publication of the genome of a closely related species, *Adineta vaga* [17], revealed a similar level of HGT, with 8 % of predicted genes of putative foreign origin. Based on the presence of these foreign genes in the same degenerate tetraploid state as observed in the rest of the genome, Flot et al. [17] predict that at least 20 % were incorporated into the genome of the common ancestor of extant bdelloid species. However, the frequency of inter-kingdom HGT events and their contribution to adaptation to different environments occupied by bdelloids remains to be determined.

Two major features of bdelloid life-history may contribute to their unusually high number of horizontally acquired genes. First, many bdelloids are extremely tolerant of desiccation [18]. It has been suggested that foreign genes might be incorporated into bdelloid genomes during cycles of desiccation and rehydration, during which DNA is broken and repaired [19–21]. Desiccation also causes cell membranes to become leaky [22, 23], thus removing an important physical barrier to exogenous DNA uptake. The combined effect of membrane porosity and chromosome breakage during desiccation could overcome obstacles for DNA exchange, frequently cited as a barrier to eukaryotic HGT. Second, bdelloids are known as ancient asexuals that lack normal meiosis [24]; indeed, analysis of the genome of *A. vaga* shows that its chromosomes are organized in such a way as to make meiotic pairing difficult [17], which by removing the need for chromosome pairing during meiosis could eliminate another obstacle to HGT usually present in eukaryotes. Recent work found evidence contrary to expectations of strict clonality within a bdelloid species [24], but it remains unclear whether that constitutes atypical meiosis or some other form of genetic exchange. It is also plausible that recombination could occur within bdelloid populations through the same mechanism as inter-kingdom HGT. While the exact nature of bdelloid reproduction and inheritance remains to be resolved, it is clear that they do not reproduce by conventional sex and meiosis.

If bdelloids do have far lower rates of sexual exchange and recombination than typical for animals, horizontally acquired genes might provide some compensation for the expected lack of genetic novelty, in a similar way that HGT facilitates adaptive shifts in prokaryotes [2]. The impact of HGT on bdelloid fitness would depend on the frequency of this process amongst bdelloid species. If the majority of foreign genes were acquired before the divergence of extant bdelloid species, uptake of novel genes cannot have contributed to the adaptation of species to new habitats. Frequent and ongoing acquisition in diversifying lineages, however, would be consistent with the role of uptake of foreign genes in adaptive divergence.

We addressed these questions by sequencing the transcriptomes of four species from the genus *Rotaria*. Unlike most bdelloids, approximately 80 % of the 24 described *Rotaria* species are fully aquatic [25], although five described species are found in terrestrial habitats. It is therefore possible to analyze species that exist in a range of habitats exposed to different desiccation frequencies. Of the four species sequenced here, *Rotaria magnacalcarata* and *Rotaria socialis* live as obligate epibionts on the fully aquatic waterlouse *Asellus aquaticus* [26]; these species therefore live permanently in freshwater. *Rotaria sordida* lives on terrestrial moss [25] and is likely to undergo frequent cycles of desiccation and rehydration, whereas *Rotaria tardigrada* is found mostly in small ephemeral pools of water and therefore also experiences desiccation, but perhaps less frequently. We investigate whether recent HGT has contributed to the genetic divergence of these closely related species and compare this to the more distantly related *Adineta* species of previous studies. Do species differ in their complement of foreign genes, consistent with a role for HGT in divergence, or did they inherit a shared set of foreign genes from a common ancestor? Taking advantage of the range of lifestyles, we also examine whether HGT has occurred more frequently in the two species living in desiccating habitats, as predicted by the DNA repair hypothesis.

## Results

### Sequencing and assembly

Illumina reads of *R. magnacalcarata*, *R. socialis*, *R. sordida* and *R. tardigrada* transcriptomes were assembled using the Trinity assembler into libraries containing 60,542, 71,102, 84,989 and 121,350 transcript contigs, with a mean length of 732, 722, 830 and 714 bp, respectively. Very low expression transcripts were excluded from further analysis (fragments per kilobase of transcript per million mapped reads (FPKM) <1 and percentage of expression for a given transcript compared with all expression from that Trinity component (IsoPct) <1), leaving 37,985, 39,937, 54,726 and 68,840 transcripts, respectively (Additional file 1: Table S1). The *A. ricciae* transcriptome assembly from Illumina reads [15] contained 61,219 transcripts. Of 12 mitochondrial genes commonly identified in bdelloid samples [27], 11 were identified in all four *Rotaria* transcriptomes, along with both ssu rRNA and lsu rRNA genes. Around half the transcripts yielded at least one significant basic local alignment search tool (BLAST) hit (e-value ≤1e-05) when compared to the UniProtKB database (47 % in *A. ricciae*, 48–51 % in *Rotaria* species). Overlap between *Rotaria* and *Adineta* transcriptomes is just under half: between 44.4 % and 48.4 % of transcripts of each *Rotaria* species in turn returned BLASTN matches with the *A. ricciae* transcriptome at e-value ≤1e-10, whereas between 85.6 % and 91.6 % of transcripts in each *Rotaria* transcriptome have a homologous transcript in another *Rotaria* sample with BLASTN match and e-value ≤1e-10. Transcripts found in more *Rotaria* species are longer and have a higher average expression level (Additional file 1: Table S2). Assemblies varied in terms of copy number: *R. sordida* and *R. tardigrada* samples have a higher proportion of genes with more than one alternative splice variant (25.4 % and 19.1 %, respectively) relative to *R. magnacalcarata* and *R. socialis* (13 % and 12.1 %, respectively; proportion test, chi-square test (chi-sq) = 5,952.8, df = 3, *P* <0.0001).

#### Proportion of putative foreign genes in *Rotaria* species

**Fig. 1:**
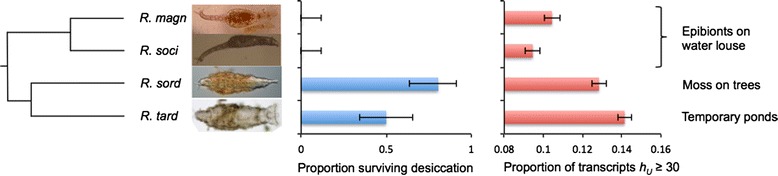
Desiccation tolerance and the proportion of horizontally transferred genes in four species of *Rotaria*. Left, blue: the proportion of individuals surviving desiccation and rehydration per species. Error bars show 95 % binomial confidence intervals. Right, red: the proportion of foreign genes with *h*_*U*_ *≥*30 with respect to the total number of transcripts analyzed per species. Note that the axis starts at proportion of 0.08, not 0, in order to display variation among species

Putative foreign genes were identified using the HGT index (*h*_*U*_ = bitscore of best non-metazoan match in UniProtKB – bitscore of best metazoan match in UniProtKB) as described by Boschetti et al. [15]. Genes with *h*_*U*_ ≥30 have a much closer match to a non-metazoan protein than to a metazoan protein and were designated as foreign. Of the 20,852, 22,130, 29,770 and 37,071 transcripts in *R. magnacalcarata*, *R. socialis*, *R. sordida* and *R. tardigrada*, respectively, with at least one hit in UniProtKB (e-value <1e-05 and with transcript length >200 bp), 10.4 % of *R. magnacalcarata*, 9.5 % of *R. socialis*, 12.8 % of *R. sordida* and 14.1 % of *R. tardigrada* transcripts were identified as foreign in origin (*h*_*U*_ ≥30; 2,176, 2,090, 3,821 and 5,242 transcripts, respectively; Fig. 1, right panel). These numbers equal or exceed the 9.7 % of *A. ricciae* transcripts with *h*_*U*_*≥*30 [15]. The estimates are conservative for several filtering criteria. For example, decreasing the e-value required for inclusion to 1e-30 suggests 12.2 %, 11.1 %, 14.5 % and 16.2 % of transcripts are foreign, respectively. Similarly, raising the minimum transcript length to 600 bp leaves 11.5 %, 10.5 %, 13.3 % and 15.4 % of transcripts, respectively, with *h*_*U*_ ≥30 (Additional file 1: Table S3). Changing these parameters did not change the relative frequencies between species. Likewise, changing the *h*_*U*_ threshold used to define a transcript as foreign did not affect the relative proportions of HGT between species (19.2 %, 18.7 %, 23.1 % and 26.1 % of transcripts have *h*_*U*_ ≥1 in turn; 7.9 %, 7.0 %, 9.6 % and 10.5 % have *h*_*U*_ ≥50). Around half of all transcripts were excluded from analysis because they failed to match any genes in UniProtKB (e-value <1e-05), preventing the identification of their likely product. If these transcripts contained no foreign genes, this would decrease the estimated frequencies of HGT to 5.7 %, 5.2 %, 7.0 % and 7.6 % in each species in turn, again not impacting the relative frequencies among species.

#### HGT and desiccation tolerance in *Rotaria*

The proportion of foreign genes (*h*_*U*_*≥*30) differs significantly among *Rotaria* species (proportion test, chi-sq = 359.07, df = 3, *P* <0.0001). *R. tardigrada* has a higher proportion of HGT (0.141 ± 0.004) than *R. sordida* (0.128 ± 0.004, chi-sq = 24.02, df = 1, *P* <0.0001), which in turn has a higher proportion than *R. magnacalcarata* and *R. socialis* (0.104 ± 0.004, chi-sq = 67.21 and 0.095 ± 0.004, chi-sq = 143.21, respectively, both df = 1, *P* <0.0001). *R. socialis* has a slightly lower proportion of HGT than *R. magnacalcarata* (chi-sq = 11.47, df = 1, *P* = 0.0007). We confirmed expected differences in desiccation tolerance among species by desiccating 36 individuals per species for 5 days, then rehydrating and scoring survival. The proportion of animals surviving desiccation differed significantly across species: no *R. magnacalcarata* and *R. socialis* from freshwater ponds survived, whereas 80.6 % of *R. sordida* individuals from moss on trees and 50 % of *R. tardigrada* individuals from a temporary pond recovered after dehydration (proportion test, chi-sq = 77.42, df = 3, *P* <0.0001; Fig. 1, left panel). *R. magnacalcarata* and *R. socialis* both had significantly lower survival than *R. sordida* (chi-sq = 45.27, df = 1, *P* <0.0001) and *R. tardigrada* (chi-sq = 21.41, df = 1, *P* <0.0001)*.* The difference in survival between *R. sordida* and *R. tardigrada* was also significant (chi-sq = 6.13, df = 1, *P* = 0.013). The two species with very low desiccation survival also had the lowest proportion of foreign genes (Fig. 1), equating to a significant, negative relationship between desiccation survival and the proportion of HGT (phylogenetic least squares regression, t = −7.74, *P* = 0.016). This pattern is consistent with the predictions of the desiccation hypothesis.

#### Foreign genes acquired before the divergence of *Adineta* and *Rotaria*

**Fig. 2:**
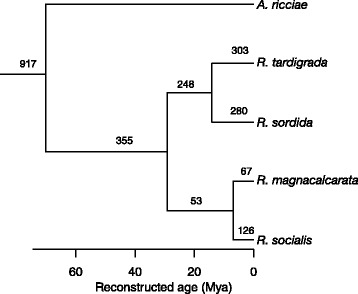
Dated phylogeny of *Rotaria* species and *A. ricciae* reconstructed from cox1, 18S and 16 nuclear genes. The tree was calibrated using published substitution rates of cox1 and 18S (see Methods). All clades have posterior probability support values = 1.0. The number of MCL orthologs unique to each species and clade are shown above branches

We compared presence/absence of foreign genes in relation to the phylogenetic relationships of the *Rotaria* species (Fig. 2). Transcripts were grouped into orthologs using reciprocal BLAST hits (see Methods). More than one third of the orthologs identified as putatively foreign in each *Rotaria* species were also present in *A. ricciae* (between 34.9 % and 55.8 % of orthologs across species; Table 1). The proportion of orthologs that were present in both *Rotaria* and *Adineta* increased further when higher *h*_*U*_ thresholds were used to designate foreign orthologs (Additional file 1: Table S4). Around half of these shared orthologs were present in all five species (Additional file 1: Table S4); the rest were absent in one or more *Rotaria* species. Some transcripts were present in clusters of similar copies, which might reflect paralogs, sequencing or assembly error, or variation within sampled populations. We therefore repeated the analysis by first grouping transcripts across species into clusters using the Markov clustering algorithm (MCL; see Methods) and treating each gene cluster as a single putative HGT event. Around half of the ortholog clusters were shared by *Rotaria* and *Adineta* (48.6 % to 61.1 % across species; Table 1, Additional file 1: Table S4).

**Table 1:** The phylogenetic distribution of HGT genes. The number and percentage of foreign genes unique to species, sister species pairs, the genus *Rotaria* and shared between *Rotaria* and *Adineta*, based on two ortholog assignment methods: 1) reciprocal BLAST and 2) Markov clusters. The unbracketed numbers show presence/absence in transcriptomes. The bracketed numbers are adjusted for false negative rates estimated from BLAST matches to the *R. magnacalcarata* genome (see Methods)

Ortholog method	Species	Unique to species		Sister species		*Rotaria*		*Rotaria* + *Adineta*		Total
		Number	Percentage (%)	Number	Percentage (%)	Number	Percentage (%)	Number	Percentage (%)	
Method 1: Reciprocal BLAST	mag	76 (53)	13.1 (6.7)	27 (37)	4.6 (4.7)	154 (351)	26.5 (43.9)	324 (356)	55.8 (44.7)	581 (798)
	soc	127 (94)	22.1 (12.3)	27 (37)	4.7 (4.9)	128 (309)	22.3 (40.5)	293 (322)	51 (42.3)	575 (763)
	sor	350 (263)	29.6 (18.8)	222 (308)	18.8 (22)	129 (294)	10.9 (21)	483 (531)	40.8 (38.1)	1,184 (1,396)
	tar	497 (364)	37 (23.3)	222 (308)	16.5 (19.7)	155 (374)	11.5 (23.9)	469 (516)	34.9 (33)	1,343 (1,561)
Method 2: Markov clusters	mag	67 (46)	6.1 (3.5)	53 (53)	4.8 (4)	305 (489)	27.9 (37)	668 (735)	61.1 (55.6)	1,093 (1,322)
	soc	126 (90)	11.8 (7.2)	53 (53)	5 (4.2)	271 (422)	25.4 (34)	617 (679)	57.8 (54.6)	1,067 (1,244)
	sor	280 (201)	17.3 (11.3)	248 (247)	15.3 (13.9)	263 (421)	16.3 (23.7)	827 (910)	51.1 (51.1)	1,618 (1,779)
	tar	303 (211)	17.9 (11.3)	248 (247)	14.6 (13.3)	321 (500)	18.9 (26.9)	823 (905)	48.6 (48.6)	1,695 (1,863)

We conclude that a core set of foreign genes was present in the common ancestor of *Rotaria* and *Adineta*. We verified this conclusion with two further analyses. First, we checked the phylogenetic distribution of four foreign genes by sequencing PCR-amplified genomic loci in a wider range of species encompassing three of the four bdelloid families (Additional file 2). Three of these genes were shared by *Rotaria* and *Adineta* transcriptomes and one was unique to *Adineta*. We found that each gene was monophyletic within bdelloids and hence likely arose from a single origin. Two genes were shared in two families (Philodinidae, which includes *Rotaria*, and Adinetidae, which includes *A. ricciae*), one was found in three families (Philodinidae, Adinetidae and Habrotrochidae) and the gene that was only present in *A. ricciae* was only successfully amplified in several species from the family Adinetidae, confirming its specificity.

Second, to check further for possible lateral transfer of shared foreign genes among bdelloids, we compared the phylogenetic signal of shared metazoan and foreign genes across species. We used the 3,330 orthologs identified by reciprocal BLAST matches that were present in all five transcriptomes (3,133 metazoan and 197 foreign). In a tree containing four *Rotaria* species, rooted with *A. ricciae*, there are 15 possible topologies (Table 2). Of 3,133 “shared metazoan orthologs”, 57.1 % conformed to topology 15: *R. magnacalcarata* and *R. socialis* monophyletic, and *R. sordida* and *R. tardigrada* monophyletic: ((mag,soc), (sor,tar)). This match relationship was reported by Fontaneto et al. [28] based on cox1 and 28S sequences and is supported by cox1 data from the transcriptomes (Fig. 2). Among 197 “shared foreign orthologs”, 65.5 % also had topology 15. Other topologies were also supported for a number of transcripts; for example, topology 1 (((mag,soc),sor),tar) was supported in 14.62 % metazoan and 14.21 % foreign shared orthologs. Foreign gene trees were no less consistent with current understanding of *Rotaria* relationships than native genes, and in fact showed less variation in topology than metazoan genes (proportion test, proportion of gene trees with topology 15 versus other topologies, chi-sq = 4.95, df = 1, *P* = 0.026). Shared horizontally acquired genes have diverged at a similar rate to their native counterparts, most genes were under purifying selection and the proportion of genes under positive selection is comparable (Additional file 3).

**Table 2:** Topologies supported by orthologous genes

Topology number	Topology (Newick format)	Metazoan genes		Foreign genes	
		Number	Percentage (%)	Number	Percentage (%)
1	(((mag,soc),sor),tar);	458	14.62	28	14.21
2	(mag,((soc,sor),tar));	2	0.06	2	1.02
3	((mag,(soc,sor)),tar);	1	0.03	1	0.51
4	(((mag,sor),soc),tar);	4	0.13	4	2.03
5	((mag,sor),(soc,tar));	1	0.03	1	0.51
6	(((mag,soc),tar),sor);	468	14.94	23	11.68
7	(mag,((soc,tar),sor));	1	0.03	0	0.00
8	((mag,(soc,tar)),sor);	2	0.06	0	0.00
9	(((mag,tar),sor),soc);	2	0.06	0	0.00
10	((mag,tar),(soc,sor));	0	0.00	0	0.00
11	(((mag,tar),soc),sor);	3	0.10	0	0.00
12	(mag,(soc,(sor,tar)));	191	6.10	9	4.57
13	((mag,(sor,tar)),soc);	209	6.67	8	4.06
14	(((mag,sor),tar),soc);	0	0.00	0	0.00
15	((mag,soc),(sor,tar));	1,790	57.13	129	65.48

#### Foreign genes acquired after the divergence of *Rotaria* from *Adineta*

Of the foreign gene clusters found in *Rotaria* but not shared with *A. ricciae*, 71.8 %, 60.2 %, 33.2 % and 36.8 % of those in *R. magnacalcarata*, *R. socialis*, *R. sordida* and *R. tardigrada* in turn were also present in at least one non-sister *Rotaria* species (e.g. *R. socialis* transcripts also found either in *R. sordida* or *R. tardigrada*; Table 1). *Rotaria* species experiencing more frequent desiccation have a higher proportion of unique genes. Foreign gene clusters shared by but unique to *R. sordida* and *R. tardigrada* are five times more abundant than genes shared by but unique to the sister species of *R. magnacalcarata* and *R. socialis* (Table 1). The number of foreign gene clusters unique to single species also varies: 67, 126, 288 and 303 clusters are unique to *R. magnacalcarata*, *R. socialis*, *R. sordida* and *R. tardigrada*, respectively. The percentage of genes that are unique to a single species was marginally higher using reciprocal BLAST to delineate orthologs instead of MCL (Table 1) and marginally lower using stricter thresholds for assigning foreign transcripts (Additional file 1: Table S4). However, the number and proportion of genes that are unique to either single species or sister species pairs was always higher for *R. sordida* and *R. tardigrada* than for *R. magnacalcarata* and *R. socialis*.

**Fig. 3:**
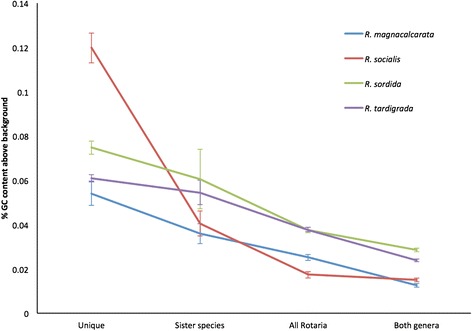
Percentage GC content of foreign genes in relation to phylogenetic distribution. GC content displayed as percentage GC content of foreign genes minus the background average GC of transcripts. Transcripts were classified as unique to species, to sister species, the genus *Rotaria* or found in both *Rotaria* and *Adineta*. GC content declines towards background levels in foreign genes with an earlier inferred entry into bdelloid genomes

The GC content of a newly acquired gene should more closely resemble that of the donor species, but over time it will approach the GC content of the genome into which it has been transferred [29, 30]; therefore, the GC content of a horizontally acquired gene should reflect the time since acquisition. The mean GC content of *R. magnacalcarata*, *R. socialis*, *R. sordida* and *R. tardigrada* transcripts retained for HGT detection analysis is 33.0 %, 33.0 %, 30.7 % and 32.1 %, respectively. In all species, GC content is most different from the background transcriptome in foreign transcripts that were only identified in a single species, and decreases significantly towards the background GC content for the species as the number of species that share the transcript increase (for *R. magnacalcarata*, *R. socialis*, *R. sordida* and *R. tardigrada* transcripts, Spearman’s rho = −0.14, −0.18, −0.20 and −0.18, respectively, all *P* <0.0001; Fig. 3). This supports the supposition that unique transcripts were acquired more recently.

### Validating the distribution of foreign genes using a draft whole genome assembly

In principle, genes absent from a particular transcriptome might be present in the species’ genome but simply not expressed at the time of RNA sampling. To examine this possibility, we searched for foreign genes in a draft genome assembly of one species, *R. magnacalcarata*. We estimated that the genome assembly was 87 % complete based on representation of 248 core proteins expected across a wide range of organisms [31]. BLAST results confirmed the transcriptome results: 79 % of foreign gene clusters inferred to be unique to *R. magnacalcarata* were found in its genome scaffolds (Additional file 1: Table S5). A further four gene clusters matched to unscaffolded genome contigs taking the recovery rate to 85 %, which is within the margin of error expected based on the completeness of the genome assembly. In contrast, only 8.7 %, 6.8 % and 8.3 % of foreign genes inferred to be unique to *R. socialis*, *R. sordida* and *R. tardigrada*, respectively, were found on *R. magnacalcarata* genome scaffolds. The false negative rate for unique foreign genes is therefore less than 10 %. Similarly, 94 % of foreign genes shared by *R. magnacalcarata* and *R. socialis* were present in *R. magnacalcarata* genome scaffolds compared with only 6 % of those defined as shared by *R. sordida* and *R. tardigrada*. Assuming a similar false negative rate for all transcriptomes would lead to a decrease in the numbers and proportions of unique genes relative to shared genes, but the differences between the two pairs of sister species would remain (shown in brackets in Table 1).

**Fig. 4:**
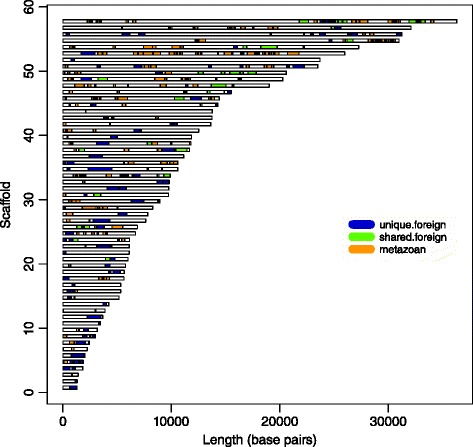
The distribution of foreign genes unique to the transcriptome of *R. magnacalcarata* across scaffolds of the draft assembly of the *R. magnacalcarata* genome. Scaffolds are sorted in order of length. Blue, foreign transcript unique to *R. magnacalcarata*; green, foreign gene shared with other rotifer species; orange, native gene (i.e. metazoan)

It is also possible that foreign genes represent contamination, for example by the RNA or DNA of a commensal non-metazoan. In fact, 71 % (41/58) of the foreign genes unique to *R. magnacalcarata* found on genomic scaffolds were on a scaffold that also included a native transcript (i.e. metazoan; Fig. 4). We searched GenBank for matches to the remaining scaffolds that contained a foreign gene but no native genes, to check whether they might be wholly non-metazoan. Of 17 scaffolds, three yielded a match with *A. vaga* (i.e. another bdelloid), two yielded matches to other metazoa (*Mus musculus* and *Lottia gigantea*), two to prokaryotes, one to fungi but only over short fragments representing the foreign gene itself (with low pairwise identities ranging from 66 % to 77 %), and nine yielded no matches at all (BLASTN, e-value ≤1e-10; Additional file 1: Table S6). Thus, we found no evidence that these scaffolds derive from contaminating, non-metazoan sequences.

### Rate of HGT across species

**Fig. 5:**
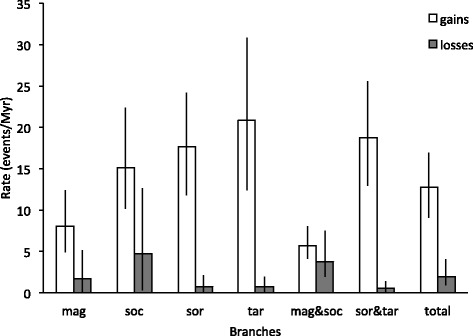
Average rates of gain and loss of foreign orthologs along the branch leading to each species, within the two clades of sister species (including stem and terminal branches) and across all *Rotaria*. MCL clusters corrected for false negative rates inferred from analysis of the *R. magnacalcarata* genome were used to estimate gains and losses; values for other versions of the analysis are in Additional file 1: Table S7. Error bars indicated 95 % HPD across BEAST trees also incorporating Poisson errors in the rate process (see Methods)

We reconstructed a dated phylogeny of the *Rotaria* species and *A. ricciae* (Fig. 2). The most recent common ancestor of *Rotaria* dates to 29 million years ago (Mya; 23.6–35.7, 95 % highest posterior density (HPD)), whereas *R. sordida* and *R. tardigrada* diverged 14.2 Mya (11.4–17.3) and *R. magnacalcarata* and *R. socialis* 6.9 Mya (5.6–8.5). These dates rely on general substitution rates of *cox1* and 18S rDNA for animals and so should be regarded as approximate. By reconstructing parsimony unambiguous gains and losses, we estimate an overall rate of gain of horizontally transferred genes of 12.8 (9.1–16.9, 95 % HPD) genes and rate of loss of 2.0 (0.9–4.1, 95 % HPD) genes per lineage per million years (Fig. 5, Additional file 1: Table S7). The rates of gain and loss on individual species branches had overlapping confidence intervals, although *R. magnacalcarata* had around half the rate of gain of the other species. However, comparing the rates of gain and loss between the two pairs of sister species (including stem and species branches), there was a threefold higher rate of gain and sixfold lower rate of loss in the *R. sordida* and *R. tardigrada* clade than in the *R. magnacalcarata* and *R. socialis* clade.

### Origin of foreign transcripts

Of the complete set of foreign transcripts, transcripts of putative eubacterial origin comprise on average 40.7 % of transcripts, archaeal sequences only comprise 1.5 % of transcripts, 19.2 % most closely resemble fungal proteins, 10.0 % of transcripts most closely resemble plant proteins and 28.7 % of transcripts most closely resemble protist proteins. Species differences are not large except that more of the foreign gene transcripts unique to *R. socialis* have their best BLASTX hit in protists than in the other species (chi-sq = 154.58, df = 1, *P* <0.0001): 59.7 % compared to 12.8 % in *R. magnacalcarata*, 11.3 % in *R. sordida* and 15.7 % in *R. tardigrada*. These 79 transcripts belong to 79 Markov clusters based on sequence similarity, so the high number is not due to post-acquisition proliferation of a few horizontally acquired genes. A BLASTX search of transcripts revealed repeated matches in *Paramecium tetraurelia* (26 transcripts), *Tetrahymena thermophila* (29 transcripts) and *Ichthyophthirius multifiliis* (14 transcripts). These are all alveolates from the class Oligohymenophorea, and the latter two both from the order Hymenostomatida. In principle, this could be the result of RNA contamination from a protist accidentally harvested from the waterlouse host alongside *R. socialis*. However, all of these transcripts differ greatly from any sequenced protist gene (all BLASTN hits >1e-05) and so any contaminating protist would have to be very divergent from any sequenced protist. Furthermore, a BLASTN search of all *R. socialis* foreign transcripts identified no hits with e <1e-05 to alveolate ribosomal RNA. If the 69 putatively alveolate genes were the result of contamination, it is likely that rRNA would also have been identified; its absence fails to support the contamination hypothesis.

### Functional annotation of foreign transcripts

*Rotaria* foreign transcripts were annotated with Gene Ontology (GO) terms [32] (identifying function for all classes of proteins) and Enzyme Commission (EC) numbers (which are restricted to enzymes). Of the 59.2 % of foreign transcripts that were assigned a GO annotation, most are enzymes as they were assigned an EC number as well (933/1,372, 68 % of *R. magnacalcarata*; 869/1,294, 67.2 % of *R. socialis*; 1,661/2,389, 69.5 % of *R. sordida*; and 1,887/2,837, 66.5 % of *R. tardigrada* transcripts also had an EC number). Enzymes with an EC number represent a significantly higher proportion of ancestral transcripts than either transcripts common to sister species or unique transcripts (44.1 % versus 34.9 %, chi-sq = 61.43, df = 1, *P* <0.0001). Many of the enzymes are involved in general metabolism, especially metabolism of carbohydrates, lipids and proteins (Table 3). These include enzymes for catabolism of plant (cellulose, xylans and pectin), fungal (chitin) and bacterial (peptidoglycans) cell walls, which might contribute to improved digestion or energy extraction from food. Some of these enzymes have been acquired by particular species or pairs of sister species but the majority are ancestral. Enzymes matching to stress responses are also prevalent, mainly as transcripts unique to particular species (Table 3). Curiously, *R. socialis* has acquired enzymes apparently linked to water deprivation (despite its desiccation intolerance), heat stress and osmotic stress (17 out of 46 orthologs annotated to GO:0006950 “response to stress” and 4 out of 6 orthologs annotated to GO:0009651 “response to salt stress” are unique to *R. socialis*; Fisher’s exact test, *P* = 0.002 and *P* = 0.000, respectively; Additional file 1: Table S8); there is nothing known in its habits to suggest it would experience these conditions more frequently than the other species. Some other classes of enzymes were significantly over-represented in particular species that had general annotations that were harder to link to potential ecological differences (Additional file 1: Table S8). For example, *R. socialis* had an excess of foreign genes involved in protein transport compared to other species. *R. sordida* had an excess of enzymes with monooxygenase activity and of those binding NADP, whereas *R. tardigrada* had an excess of genes with oxidoreductase activity acting on CH-OH donors and of those binding ATP.

**Table 3:** Categorization of foreign transcripts according to their potential role in macromolecular catabolism or stress and with respect to their distribution across species

Functional category		mag	soc	sor	tar	mag&soc	sor&tar	rot	All
Polysaccharide	Pectin	1							1
	Cellulose					2			16
	Chitin		1						24
	Xylan				1		3		5
	Glycan	2			3				37
	Glycoside		2	5	4		18	18	89
	Starch								22
Lipid	Lipase		1	2	3		8	32	72
	Esterase			3			2	5	106
Protein	Protease		2	1	6		11	22	35
Stress	Starvation			1			1		
	Oxidative		1	1	2			2	3
	Heat			1					
	Water		2						
	Fungus		1						
	Virus						2		
	Osmotic		4	1				1	2
	Cold			3					
	Heat		6	1					1
	DNA repair			1					4

To test further for differential presence of foreign genes related to water stress and desiccation, we searched for annotated functions matching those of genes potentially associated with desiccation tolerance in *A. ricciae*. We expected that these genes might have been differentially lost in the desiccation intolerant commensal species. Among the functions suggested to be differentially regulated during desiccation of *A. ricciae* [16, 33], seven (amidase, gluconolactonase, inorganic phosphate transporter, ubiquitin-conjugating enzyme, IBR-containing protein, DNAj-related protein and cellulase) were found among the *Rotaria* foreign orthologs; a further one, catalase, was found in *R. sordida* but clustered in a native ortholog. We also searched for enzymes associated with glutathione and trypanothione metabolism, which are considered powerful antioxidants, and which could protect bdelloid cells against oxidative damage during desiccation [15]. Of 17 orthologs present in the *Rotaria* transcriptomes, three were judged to have been lost in *R. magnacalcarata* and *R. socialis*, which is a higher ratio than expected from background rates of loss of foreign orthologs in those species (94/3,514 orthologs, Monte-Carlo simulation, chi-sq = 14.7, *P* = 0.0094). This suggests, therefore, that foreign genes potentially associated with protection from desiccation damage have been lost in the commensal species.

## Discussion

We found that 9.5–14.2 % of transcripts had *h*_*U*_ ≥30 in the four *Rotaria* species examined. To date, calculation of the portion of foreign genes in bdelloid genomes or transcriptomes has focused on species from a single genus, *Adineta*, within the family Adinetidae and on a couple of genomic regions for one species of Philodinidae [15, 17]. Finding evidence of a high proportion of HGT in species from multiple families demonstrates that abundant HGT is not unique to *Adineta* species, with their presumed frequent cycles of desiccation and rehydration, but is a feature of bdelloid rotifers more widely, even those from permanently aquatic habitats.

Nearly half of foreign genes expressed by *R. magnacalcarata*, *R. socialis*, *R. sordida*, *R. tardigrada* and *A. ricciae* are common to both families Philodinidae and Adinetidae. These genes are most parsimoniously assumed to have been present in the common ancestor. The alternative explanation of substantial rotifer-to-rotifer HGT of foreign genes after the divergence of bdelloid species seems unlikely; gene trees based on foreign genes and on metazoan genes are congruent, and where foreign genes were examined in more detail, monophyly of these genes within bdelloids points to a single uptake event of each horizontally acquired gene. Furthermore, the GC content of shared foreign genes is much closer to that of the metazoan bdelloid transcripts than the GC content of species-specific foreign genes, consistent with their presence in bdelloid genomes for a longer time [29, 34]. These genes were therefore acquired before the divergence of bdelloid families. Foreign genes common to *Adineta* and *Rotaria* species present a rare example of a large number of foreign genes being acquired and then maintained in a eukaryotic genome through millions of years and multiple speciation events. The acquisition of many enzymes with novel functions might have enhanced biochemical diversity in bdelloids, allowing them to perform some of their unusual functions, such as desiccation tolerance [15].

Alongside foreign genes identified as common to *Rotaria* and *Adineta* species, we also identified genes common to the genus *Rotaria*, genes common to sister species only, and those unique to individual species. The number of foreign genes in any bdelloid genome is the result of both gains and losses over time, so the higher number of foreign genes in some species could conceivably be due to either or both of these processes: more gains in some species or more losses in others. However, the average GC content of foreign genes becomes more similar to that of the background bdelloid genome the more species that gene is shared by. This finding implies that genes shared by fewer species have been gained more recently by these species, rather than having been differentially lost from the others [29]. These more recently acquired genes demonstrate that HGT is an ongoing process in multiple bdelloid species, and could potentially provide genetic variation underlying species differences. However, the high proportion of ancient foreign genes, along with the slow rate of gain of 12.8 genes per million years highlights the fact that inter-kingdom HGT in bdelloids can by no means be considered as a “replacement for sex”. We could not quantify uptake of DNA from other metazoans here because of insufficient sampling of closely related metazoan genomes to identify transfer; it is likely that bdelloids also take up metazoan DNA and this might occur at a faster rate than inter-kingdom HGT.

*Rotaria* species with higher desiccation tolerance (*R. sordida* and *R. tardigrada*) have more unique expressed genes than those unable to survive desiccation (*R. magnacalcarata* and *R. socialis*). Foreign genes shared by and unique to *R. sordida* and *R. tardigrada* are nearly five times more abundant than genes shared by *R. magnacalcarata* and *R. socialis*, and accumulated at a faster rate. The relationship between HGT and desiccation tolerance/frequency needs to be examined in more bdelloid species encompassing multiple shifts in habitat type, but findings from within *Rotaria* provide initial support for the hypothesis that the rate of HGT is increased by cycles of desiccation and rehydration in bdelloid rotifers [14, 20, 21]. This unusual feature of bdelloid ecology could provide part of the explanation for why bdelloid rotifers have such an extraordinarily high level of HGT for metazoans. However, the two fully aquatic species both show evidence of HGT since their divergence. Either complete desiccation is not a strict requirement of HGT, or these species might still recover from desiccation rarely or in particular desiccating conditions.

The foreign genes identified in bdelloids have been acquired from multiple different kingdoms [14, 15]. In *Rotaria* we find that the majority come from eubacteria followed in descending order by protists, fungi, plants and archaea. Similar frequencies were found in *A. ricciae*, although that species had relatively more prokaryotic origins and half the frequency of protist genes [15]. The high representation of bacterial genes in eukaryotic HGT is likely to be related to bacterial size and ubiquity, which makes them more likely to live alongside, be food for, or be pathogens of, multicellular eukaryotic species. In the laboratory the most successful food sources for bdelloid rotifers are bacteria and fungi [35]. There are two possible explanations for the finding of over-abundance of protist genes in *R. socialis*: contamination of extracted RNA by an alveolate protist or a large uptake of protist genes into the *R. socialis* genome after the divergence from *R. magnacalcarata*. Contamination is unlikely because of the lack of protist ribosomal genes in the transcriptome. *R. magnacalcarata* and *R. socialis* both live on the ventral side of the waterlouse: *R. socialis* is found at the sides, close to the legs, whilst *R. magnacalcarata* is distributed towards the anterior median of the host animal [26]. Cook and Chubb [36] demonstrated that peritrich protists (class Oligohymenophorea) make up nearly 90 % of the epifauna of *Asellus aquaticus*, and their scanning electron microscope (SEM) analysis showed that the majority of these species are found to the periphery and on the legs of the ventral side of the waterlouse. *R. socialis* might have taken up protist genes thanks to its greater proximity to epibiont protists relative to *R. magnacalcarata*, although mechanisms of HGT remain obscure for all bdelloids.

To be maintained in the genome of all these species since the divergence of *Rotaria* and *Adineta* species, the foreign genes common to all species must have had little negative fitness impact on the individuals. The presence of foreign genes in transcriptomes indicates that these genes are expressed, although in line with the hypothesis of Park and Zhang [37] that highly expressed genes are not favoured for HGT, they are expressed at a slightly lower level in comparison to total transcripts (mean HGT FPKM = 21.1; mean total FPKM = 26.3; t = −2.9, *P* = 0.0037).

Consistent with previous findings in *A. ricciae* [15] and studies in prokaryotes [38], we found that a high proportion of foreign genes in *Rotaria* govern metabolic functions. A significantly higher percentage of foreign transcripts acquired before the divergence of *Adineta* and *Rotaria* species were annotated with EC numbers in comparison to more recently acquired foreign genes, suggesting that in *Rotaria* horizontally acquired genes encoding biochemical functions may be more likely to be retained than other genes. Foreign genes have contributed hundreds of novel enzymes associated with a range of annotated functions of potential ecological importance. In particular, there are many enzymes involved in the catabolism of macromolecules that might play a role in extracting energy from food sources. Diets of bdelloids are difficult to observe directly in the wild and tools for genetic knockouts in bdelloids are still being developed, but future work could test for differential expression of shared and unique foreign enzymes when cultured in the laboratory on different diets.

We found no evidence for greater acquisition of stress-related genes in the desiccating species – on the contrary, *R. socialis* has acquired an excess of water, salt and heat stress genes. It is possible that these genes perform different functions than in their donor organism or they might protect against other forms of stress such as freezing or anoxia, which are known to be experienced by the waterlouse. Some of the foreign genes with no annotation or unclear affiliation to stress responses in *R. sordida* and *R. tardigrada* might also play a role in desiccation tolerance and, again, future work could test for differential expression of unique enzymes under different stress conditions. There was evidence, however, for differential loss of ancestral foreign genes in the two commensal species, including genes involved with the production of antioxidants that are thought to protect against oxidative damage during desiccation [13].

## Conclusions

Many of the horizontally acquired genes identified in bdelloid rotifers are likely to have been inherited from the common ancestor of all bdelloid species, but a significant subset of foreign genes have been acquired more recently and are unique to specific lineages, especially those from desiccating habitats. While genes acquired in a common ancestor can have had no role in providing genetic novelty between bdelloid species, genes acquired post-divergence of bdelloid taxa have contributed differences in enzyme complements. Further work is now needed to identify the contribution of native genes versus foreign genes to adaptive trait differences between these species. Differential acquisition of foreign genes is known to contribute to adaptation and speciation in prokaryotes [7], and our results are consistent with a similar role in this clade of animals.

## Methods

### Rotifer sampling

*Rotaria magnacalcarata* and *Rotaria socialis* were isolated from waterlice sampled from the pond in the Japanese Garden at Silwood Park Campus, Ascot, UK. *Rotaria sordida* was isolated from moss at the bottom of a single tree on the Silwood Park Campus. *Rotaria tardigrada* was isolated from silt at the bottom of the pond in the pond field at Silwood Park Campus (Table 4). Between 140 and 300 individuals were isolated for each RNA extraction, and two extractions were combined to produce each final cDNA sample for sequencing. RNA was isolated from population samples because the species cannot be easily cultured in the laboratory. Bdelloid morphological species often contain many cryptic species, but we selected populations thought to comprise only a single genetic evolutionarily significant unit (ESU) based on previous sequencing of cytochrome oxidase I and 28S [28]. After filtering out extremely rare transcripts (sequencing and assembly), only single copies of *cox1* were present in each transcriptome.

**Table 4:** Details of samples collected for sequencing

Species	RNA sample	Date sampled	Location sampled	Habitat	Number of individuals
*Rotaria socialis*	Rsoc1	2 September 2011	Japanese pond, Silwood Park Campus, Ascot, UK	Living on waterlice (*A. aquaticus*) amongst leaf litter in the pond	250
(Kellicott 1888)	Rsoc2	15 September 2011	51.407 N, 0.640 W		250
*Rotaria magnacalcarata*	RMc3	21 September 2011	Japanese pond, Silwood Park Campus, Ascot, UK	Living on waterlice (*A. aquaticus*) amongst leaf litter in the pond	250
(Parsons 1892)	RMc4	30 September 2011	51.407 N, 0.640 W		250
*Rotaria sordida*	Rsord5	16 October 2012	Silwood Park Campus, Ascot, UK	Moss at bottom of tree	300
(Western 1893)			51.408 N, 0.642 W		
*Rotaria tardigrada*	RTard4	12 November 2012	Pond in pond field, Silwood Park Campus, Ascot, UK	Silt at the bottom of the pond	140
(Ehrenberg 1832)			51.412 N, 0.642 W		

### RNA extraction and cDNA synthesis

RNA was extracted from each sample using the RNeasy Mini Kit (Qiagen, Venlo, Netherlands). RNA purity and concentration were checked using a NanoDrop spectrophotometer (NanoDrop, Wilmington, DE, USA). For both *R. socialis* and *R. magnacalcarata*, a pair of RNA samples were combined and further purified using ethanol–sodium acetate precipitation, to produce a more concentrated sample for input into the cDNA synthesis reaction. Oligo(dT)-primed cDNA from *R. socialis* and *R. magnacalcarata* samples was prepared using the Clontech/Takara SMARTer PCR cDNA Synthesis Kit and an Advantage 2 PCR Enzyme System (Clontech, Mountain View, CA, USA) using SuperScript II Reverse Transcriptase (Invitrogen, Carlsbad, CA, USA). Oligo(dT)-primed cDNA from *R. sordida* and *R. tardigrada* samples was prepared using the Clontech/Takara SMARTer PCR cDNA Synthesis Kit and an Advantage 2 PCR Enzyme System. cDNA samples were not normalized prior to sequencing because of the potential loss of material from relatively small starting amounts of RNA. We instead opted for high sequencing effort.

### Transcriptome sequencing and assembly

cDNA libraries were prepared and sequenced by The Eastern Sequence and Informatics Hub (University of Cambridge, Cambridge, UK). Illumina sequencing was performed in a single HiSeq lane for one cDNA library per species (*R. magnacalcarata*, *R. socialis*, *R. sordida* and *R. tardigrada*; Illumina, San Diego, CA, USA). These were assembled with the Trinity assembler [39]. A subset of very low expression transcripts, with IsoPct (per component expression level) <1 and FPKM <1 were excluded from further analysis. The number of transcripts retained at each step of processing is recorded in Additional file 1: Table S1. Sequence data are available in GenBank (accession numbers GDRE00000000, GDRD00000000, GDRH00000000 and GDRK00000000). *Rotaria* transcriptomes were compared to the published transcriptome for *A. ricciae* [15].

To check for the possibility of variation in copy number between transcriptomes, Trinity assemblies were used to identify possible alternative splice variants and paralogs within sequence groups. We compared the frequency of both alternative splice variants and paralogs across the four *Rotaria* assemblies. The number of transcript groups containing different numbers of paralogs did not vary significantly between species.

### Genome sequencing and assembly

*Rotaria magnacalcarata* were isolated from the same sampling location as for RNA extractions. Between 300 and 350 individuals were isolated for each DNA extraction and three extractions then combined to produce the final DNA sample for sequencing. DNA was extracted using the DNeasy Mini Kit (Qiagen). DNA concentration and purity was assessed using a NanoDrop spectrophotometer and by gel electrophoresis. Illumina TruSeq Nano libraries with 550 bp inserts were prepared by the Department of Biochemistry, University of Cambridge, UK. Sequencing was performed on four lanes of an Illumina NextSeq500, producing >160 million paired-end reads. Trim Galore! [40] and Trimmomatic [41] were used to remove Illumina adapter sequences and low quality reads from the dataset. Trimmed reads were assembled into contigs and scaffolded using SOAPdenovo2 [42]. The draft assembly was further processed using L_RNA_scaffolder [43] and GapFiller [44] automated scripts to improve scaffolding and reduce N content. We used CEGMA [45] to assess assembly completeness and QUAST 3.0 [46] to assess genome assembly metrics. The assembly had a total length of 449.6 Mb, half of the scaffolds had length >280 bp and 24 % had length >9,014 bp. Average GC content was 32.5 %, N content was 3.8 % and 97.5 % of *R. magnacalcarata* transcripts had a significant BLAST match in the genome (e-value ≤1e-05). Scaffolds that contained foreign transcripts are available from GenBank (accession number LJPC00000000).

### HGT detection

Putative foreign genes were identified using the HGT index (*h*_*U*_) and methods described in detail by Boschetti et al. [15]. All analyses were conducted in R (The R Project for Statistical Computing, http://www.r-project.org/). In brief, BLAST searches were performed using NCBI-BLAST 2.2.25+ [47], ClustalW2 (EMBL-EBI) [48] was used for sequence alignment and PhyML 3.0 [49] was used to produce phylogenetic reconstructions. The software pipeline was controlled using the “system” function in R. For each species, the set of transcripts >200 bp were compared using BLASTX to taxon-specific subsets of UniProtKB. Subsets were metazoa, eubacteria, archaea, plants, fungi and “other eukaryotes” in turn, and only proteins from complete proteomes were included (keyword: KW-0181). Sequences that did not match with at least one taxon with an e ≤1e-05 were excluded from further analysis, leaving 20,852, 22,130, 29,770 and 37,071 transcripts for *R. magnacalcarata*, *R. socialis*, *R. sordida* and *R. tardigrada*, respectively. Note that we use a less stringent e-value than typical for searches of GenBank because we searched the smaller database of UniProtKB. The HGT index (*h*_*U*_), the difference between the highest non-metazoan and the highest metazoan bit-scores, was calculated for the remaining sequences. A high value indicates a stronger match to a non-metazoan taxon than to metazoans. A threshold of *h*_*U*_ ≥30 (as used in Boschetti et al. [15]) was used to define potentially horizontally transferred genes. Transcripts with *h*_*U*_ ≥30 were divided into groups based on the kingdom with the lowest BLASTX e-value: “eubacteria”, “archaea”, “fungi”, “plants” or “protists”. Results are shown in Additional file 1: Table S9.

### Contamination check

Each transcript with *h*_*U*_ ≥30 was used as a query in a BLASTN search against the non-redundant database (nr), which consists of nucleotide sequences from GenBank, EMBL, DDBJ, PDB and RefSeq. Overall, 74 *R. magnacalcarata* transcripts, 60 *R. socialis* transcripts, 77 *R. sordida* transcripts and 44 *R. tardigrada* transcripts with close BLASTN hits (e-value ≤1e-05) were defined as potential contaminants. These transcripts were then searched for in the *A. ricciae* transcriptome using BLASTN. If a sequence returned a match in *A. ricciae* with an e-value of ≤1e-10 it was not considered a contaminant, and only the remaining five *R. magnacalcarata*, ten *R. socialis*, five *R. sordida* and nine *R. tardigrada* transcripts were excluded from analyses. Over half (16/29) of the contaminating sequences identified were bacterial, two were viral sequencing artefacts, and the remainder were dispersed between other kingdoms.

### Desiccation tolerance

A 1 cm^2^ piece of filter paper was added to each well of six 24-well plates, as a substrate for desiccating animals to attach to [50]. Individuals were sampled from the same locations as transcriptome isolates, but the following year. Each individual was isolated, washed and placed in a well with 1,500 μl water. After 1 h excess water was removed, leaving a film of water with each rotifer. Plates were left in an incubator at 20 °C. After 5 days complete desiccation was confirmed using cobalt chloride paper and water was added to each well. After 4 hours of rehydration samples were examined and individuals showing movement were scored as “survived”. Samples were re-checked after a further 4 hours to allow for slow recovery. In order to test the relationship between desiccation survival and proportion of HGT, a phylogenetic generalized least square (PGLS) model in the R package caper 0.5.2 was used [51, 52]. The percentage of HGT for each species was used as a response variable and the proportion of survival after desiccation was used as an explanatory variable in a model using the phylogeny of the four species (described below) with branch length transformations (lambda, delta and kappa) optimized by maximum likelihood. For both the response and the explanatory variable arcsine square root transformed values were used, because the original variables were proportion data-bound at the extremes.

### Between species comparisons

Orthologous genes amongst *Rotaria* species were identified using BLASTN (dc-megablast) to detect reciprocal best hits between species (e-value ≤1e-10). First reciprocal best hits between species pairs were identified, then the six pair-wise comparisons were combined and genes where reciprocal best hits were consistent across these comparisons were retained. These were used to query the *A. ricciae* transcriptome using BLASTN (dc-megablast) to identify orthologs with a homolog in *A. ricciae* (e-value ≤1e-10). In most orthologs (65.3 %) that contained at least one foreign transcript (*h*_*U*_*≥*30), all of the transcripts were identified as foreign. Among the remaining cases with a mixture of foreign and native transcripts, orthologs with more than half foreign transcripts were designated “foreign orthologs”. We checked alignments and in all these cases one or more species yielded a score just below the threshold of 30, usually associated with a much shorter transcript than in the other species. All other orthologs were designated as metazoan, i.e. native. Varying the criterion for defining mixed orthologs as foreign altered the relative proportion of shared orthologs but did not change relative differences among species (Additional file 1: Table S4). The coding region of transcripts was detected using OrfPredictor (http://proteomics.ysu.edu/tools/OrfPredictor.html) [53].

Both native and foreign genes were sometimes present in multiple copies. We therefore repeated the classification of phylogenetic distribution using a clustering approach that groups similar transcripts both within and between species. Transcripts across all species were pooled and clustered into groups based on similarity using the MCL algorithm [54, 55]. MCL identifies gene families based on graph flow theory, utilizing a MCL procedure. An all-against-all comparison was performed using BLASTN, and MCL clusters were created with e-value of 1e-10 and clustering granularity of I = 20. Repeating with e-values of 1e-5 and 1e-20 and granularities of 16 to 44 had little effect on the results (Additional file 1: Table S4). MCL led to retention of more orthologs than reciprocal BLAST because transcripts with inconsistent reciprocal matches between species were now retained as part of a larger cluster.

### Phylogenetic analyses

Orthologous genes found in all five species were aligned using ClustalW2 and trees were constructed using PhyML (GTR + I + G model, SH-like support values). Phylogenies were rooted on *A. ricciae*. To estimate divergence times between species, we first identified cytochrome oxidase 1 (*cox1*) transcripts in each *Rotaria* transcriptome (accession numbers gb|GDRE01025734.1, gb|GDRD01031279.1, gb|GDRH01017651.1 and gb|GDRK01007754.1) and in the transcriptome of *A. ricciae* (gi|425844704). Where there was more than one copy of the mitochondrial gene identified in a sample (due to population variation or sequencing error), the sequence with highest expression (FPKM) was selected for analysis. We extracted 18S rRNA sequences from each *Rotaria* transcriptome (accession numbers gb|GDRE01005169.1, gb|GDRD01030651.1, gb|GDRH01022638.1 and gb|GDRK01031536.1) and in the transcriptome of *A. ricciae* (gi|425834109). We also selected nuclear genes identified as present across all five species and for which the alignment contained <2 % gaps, yielding 15 nuclear genes (Additional file 1: Table S10). Genes were aligned using ClustalW and checked by eye. MODELTEST [56] selected the general time-reversible (GTR) model [57] with invariant sites and gamma-distributed rate variation (GTR + Γ + I) as the most likely substitution model using the Akaike information criterion. A dated phylogeny was reconstructed in BEAST version 1.8.1 [58] from the concatenated matrix, using separate GTR + Γ + I substitution models for each gene partition, a relaxed lognormal clock, constant birth rate prior, a random starting tree, 10,000,000 generations and sampling every 1,000 generations. Substitution rate calibrations were supplied for cox1 (1.76 % per million years chosen for the GTR + Γ + I model) [59] and 18S (0.02 % per million years) [60]; as in Tang et al. [51]. Trees were combined into maximum credibility trees using TreeAnnotator version 1.8.1.

We reconstructed gains that were parsimony unambiguous as those where a single gain of a foreign gene was more parsimonious than any other scenario. This equated to any gene present in a single species, or shared only by two sister species of *Rotaria*. The inverse patterns were used to infer losses. We estimated the Poisson rate of gains and losses in turn by dividing the number of events by the duration of the branch upon which they were reconstructed to occur. We repeated this across sampled BEAST trees to calculate the 95 % HPD interval. In order to account for uncertainty due to sampling, for each tree we took a sample from a Poisson distribution with mean rate of the observed number of events per unit time for that tree, so that the final HPD interval encompassed both sampling of events and uncertainty in the dates.

### GC content of foreign transcripts

The GC content of each transcript was calculated using the GC.content function in the package “ape” version 3.0-9 [52] in R (The R Project for Statistical Computing, http://www.r-project.org/). Background GC content for each transcriptome was calculated for the subset of transcripts retained for HGT detection (20,852 *R. magnacalcarata*, 22,130 *R. socialis*, 29,770 *R. sordida* and 37,071 *R. tardigrada* transcripts). This was then compared to GC content in foreign transcripts unique to each species or shared by more exclusive sets of species in turn.

### Gene annotation analysis

Foreign *Rotaria* transcripts (*h*_*U*_ ≥30) were annotated using Blast2GO version 2.6.6 [61], using default settings for mapping and annotation, except for the removal of evidence code control from the annotation step (evidence code cut-off = 1): 1,372 of 2,176 *R. magnacalcarata* HGT transcripts (63.1 %), 1,294 of 2,090 *R. socialis* HGT transcripts (61.9 %), 2,389 of 3,821 *R. sordida* transcripts (62.5 %) and of 2,837 of 5,242 *R. tardigrada* transcripts (54.1 %) were assigned at least one GO annotation. GO numbers were then compared against the EC2GO database (last modified 10 October 2013 [62]) and corresponding EC numbers were retrieved. Enrichment of GO categories was determined using the topGO library [63] in Bioconductor [64].

## Additional files

Additional file 1: Table S1. Number of reads/sequences retained at each stage of the assembly process. **Table S2.** Transcript length and read depth in relation to the number of species in which transcripts were identified. Transcripts from each *Rotaria* species in turn were used as queries in a BLASTN search of the other three *Rotaria* transcriptomes (e-value ≤1e-10). Mean transcript length and FPKM is presented in relation to the number of species transcripts were identified in. **Table S3.** Percentage of transcripts with *h*_*u*_ ≥30 with varying HGT detection thresholds. Left: percentage of transcripts with *h*_*u*_ ≥30, for decreasing e-value thresholds for accepting transcripts for analysis. Right: percentage of transcripts with *h*_*u*_ ≥30, for increasing minimum transcript length for analysis. Red, *R. magnacalcarata*; blue, *R. socialis*; green, *R. sordida*; and black, *R. tardigrada.* **Table S4.** The number of orthologs that are unique to different subclades based on presence/absence in transcriptomes for different methods of identifying orthologs and different criteria for assigning orthologs as foreign or not. **Table S5.** BLASTN matches in the *R. magnacalcarata* genome of genes inferred to be unique to particular species based on transcriptome presences/absences. **Table S6.** Best BLASTN matches of scaffolds that contain a foreign gene but no non-foreign gene in *R. magnacalcarata* in GenBank. **Table S7.** Reconstruction of numbers and rates of gains and losses of foreign genes on different branches or sets of branches on the *Rotaria* tree. Results based on two methods of assigning orthologs are shown: 1) reciprocal BLAST and 2) Markov clusters; and excluding or including correction based on *R. magnacalcarata* genome matches. **Table S8.** Results of topGO enrichment analysis for GO annotation categories that are over-represented in particular species or sets of species relative to numbers across the whole foreign ortholog dataset. Fisher’s exact tests with *P* <0.01 are shown in bold. The “Weight01” option was used to correct for significance of nested annotation terms. Only terms with more than five orthologs present in the whole dataset are shown. **Table S9.** List of foreign transcripts (*h*_*U*_ >30), results of the HGT analysis, GO annotation and matches to the *R. magnacalcarata* draft genome. **Table S10.** List of nuclear genes used in phylogenetic reconstruction. (XLSX 3668 kb) Additional file 2:
**Survey of four foreign genes in the gDNA of a selection of bdelloid species.** (DOCX 4251 kb) Additional file 3:
**Comparison of divergence and selection in native and foreign genes.** (DOCX 93 kb)
