# Supplementary material for: Horizontal gene transfer in bdelloid rotifers is ancient, ongoing and more frequent in species from desiccating habitats
Source: BMC Biol. 2015 Nov 4;13:90. doi: 10.1186/s12915-015-0202-9 (PMC4632278; doi:10.1186/s12915-015-0202-9)

**Additional file 2.** Survey of four foreign genes in the gDNA of a selection of bdelloid species.

In order to test predictions made on the basis of presence or absence in transcriptome data about the distribution of horizontally acquired genes across bdelloids more widely, we used PCR followed by Sanger sequencing to look for the presence of four foreign genes across a wide range of bdelloid species, with representatives from three of the four bdelloid families, and from a wide range of geographic locations (table 2.1).

**Table 2.1.** Samples used for sequencing of candidate genes, including country of origin where available.

| **Sample name** | **Family** | **Species** | **Country** |
| --- | --- | --- | --- |
| A558.AB1 | Adinetidae | *Adineta barbata* | Sweden |
| A558.AB4 | Adinetidae | *Adineta barbata* | Sweden |
| A545.AC2 | Adinetidae | *Adineta cuneata* | Sweden |
| A545.AC3 | Adinetidae | *Adineta cuneata* | Sweden |
| A545.AC4 | Adinetidae | *Adineta cuneata* | Sweden |
| A545.AC5 | Adinetidae | *Adineta cuneata* | Sweden |
| AG25.1 | Adinetidae | *Adineta gracilis* | UK |
| AR | Adinetidae | *Adineta ricciae* | Australia |
| BG0744_ASt1 | Adinetidae | *Adineta steineri* | Bulgaria |
| PloASt11 | Adinetidae | *Adineta steineri* | Poland |
| A661.AS1 | Adinetidae | *Adineta steineri* | Sweden |
| A661.AS2 | Adinetidae | *Adineta steineri* | Sweden |
| A661.AS3 | Adinetidae | *Adineta steineri* | Sweden |
| A661.AS4 | Adinetidae | *Adineta steineri* | Sweden |
| A661.AS5 | Adinetidae | *Adineta steineri* | Sweden |
| S09-1_ASt1 | Adinetidae | *Adineta steineri* | UK |
| A667.AT1 | Adinetidae | *Adineta tuberculosa* | Iceland |
| A667.AT2 | Adinetidae | *Adineta tuberculosa* | Iceland |
| A667.AT3 | Adinetidae | *Adineta tuberculosa* | Iceland |
| A667.AT4 | Adinetidae | *Adineta tuberculosa* | Iceland |
| A550.AT1 | Adinetidae | *Adineta tuberculosa* | Sweden |
| A550.AT2 | Adinetidae | *Adineta tuberculosa* | Sweden |
| A550.AT3 | Adinetidae | *Adineta tuberculosa* | Sweden |
| A550.AT4 | Adinetidae | *Adineta tuberculosa* | Sweden |
| A505.AV10 | Adinetidae | *Adineta vaga* | Sweden |
| A505.AV11 | Adinetidae | *Adineta vaga* | Sweden |
| A505.AV12 | Adinetidae | *Adineta vaga* | Sweden |
| A505.AV9 | Adinetidae | *Adineta vaga* | Sweden |
| 1R1a | Adinetidae | *Adineta vaga* | UK |
| JGAV4 | Adinetidae | *Adineta vaga* | UK |
| 3R1a | Adinetidae | *Adineta vaga* | Japan |
| orange WB1 | Adinetidae | *Adineta vaga* | Japan |
| D10.6 | Adinetidae | *Adineta vaga* | UK |
| 3Q1a | Adinetidae | *Adineta vaga* | USA |
| 2T34 | Adinetidae | *Adineta vaga* | USA |
| 2V4 | Adinetidae | *Adineta vaga* | UK |
| 2V5 | Adinetidae | *Adineta vaga* | UK |
| B1 | Adinetidae | *Adineta vaga* | UK |
| O1 | Adinetidae | *Adineta vaga* | UK |
| P1 | Adinetidae | *Adineta vaga* | UK |
| A734.DA1 | Philodinidae | *Dissotrocha aculeata* | Svalbard, Norway |
| A734.DA2 | Philodinidae | *Dissotrocha aculeata* | Svalbard, Norway |
| A810.PM21 | Philodinidae | *Philodina megalatrocha* | UK |
| A810.PM22 | Philodinidae | *Philodina megalatrocha* | UK |
| A811.PM13 | Philodinidae | *Philodina megalatrocha* | UK |
| A811.PM14 | Philodinidae | *Philodina megalatrocha* | UK |
| A797.PC4 | Philodinidae | *Philodina citrina* | Italy |
| A595.RMn3 | Philodinidae | *Rotaria mento* | Sweden |
| SA595.RMn4 | Philodinidae | *Rotaria mento* | Sweden |
| A595.RMn5 | Philodinidae | *Rotaria mento* | Sweden |
| A595.RMn6 | Philodinidae | *Rotaria mento* | Sweden |
| A595.RMn7 | Philodinidae | *Rotaria mento* | Sweden |
| A595.RMn8 | Philodinidae | *Rotaria mento* | Sweden |
| A708.RR8 | Philodinidae | *Rotaria rotatoria* | Sweden |
| A718.RR4 | Philodinidae | *Rotaria rotatoria* | Sweden |
| A788.RR01 | Philodinidae | *Rotaria rotatoria* | UK |
| A788.RR02 | Philodinidae | *Rotaria rotatoria* | UK |
| A788.RR03 | Philodinidae | *Rotaria rotatoria* | UK |
| A814.RR2 | Philodinidae | *Rotaria rotatoria* | UK |
| A513.RR2 | Philodinidae | *Rotaria rotatoria* | Sweden |
| A790.RR67 | Philodinidae | *Rotaria rotatoria* | Italy |
| A718.RR1 | Philodinidae | *Rotaria rotatoria* | Sweden |
| A790.RR66 | Philodinidae | *Rotaria rotatoria* | Italy |
| A801.RT03 | Philodinidae | *Rotaria tardigrada* | Italy |
| A801.RT05 | Philodinidae | *Rotaria tardigrada* | Italy |
| A801.RT04 | Philodinidae | *Rotaria tardigrada* | Italy |
| 2H1 | Habrotrochidae | *Habrotrocha ligula* |  |

BLASTN was used to search the *A. ricciae* transcriptome for foreign genes corresponding to the ones originally identified in the available genomic regions of *Adineta vaga* (Gladyshev et al., 2008). Four putatively horizontally acquired genes with successful BLASTN hits in *A. ricciae* (e ≤ 1e-05 and >40% coverage) were selected for analysis: a short-chain dehydrogenase AV10109 (gb|EU643480.1), an endo-β-xylanase AV10027 (gb|EU643475.1), a β-galactosidase AV10092 (gb|EU643479.1) and an FAD-binding monooxygenase AV10134 (gb|EU643488.1). Amplification of these genes was then attempted in all samples in table 2.1, using primer sequences shown in table 2.2. All sequences >200bp can be found in genbank, accession numbers KT756807- KT756873.

**Table 2.2:** primers for amplification of the four candidate genes.

| **Gene ID** | **Primers** | **Expected length** |
| --- | --- | --- |
| AV10092 | AV10092.1_F: TGCAACGGYCWTKCGGCTAA  AV10092.1_R: TCGGYTCGTTCGGYGTACCA | 340bp |
| AV10109 | AV10109.1_F: ACGGGACGACGAAAACAAAAACTTG  AV10109.1_R: TYGGTRTTGCTKCTKKCCACAT | 503bp |
| AV10134 | AV10134.1_F: CGCSGCRCACCAAATGCCRC  AV10134.1_R: GCGCACGACTYCCRGGTCTY | 395bp |
| AV10027 | AV10027.1_F: AGCAGCAGGTGCATTTGAGCC  AV10027.1_R: TCGTTCAGGTGCTGATCCCGC | 157bp |

Genes encoding β-galacotosidase do exist in metazoans, and a search of GenBank revealed a transcript coding for β-galactosidase in the monogonont rotifer *Brachionus plicatilis* (gi|259479143)*.* To confirm that the putatively foreign copy of β-galactosidase (AV10092) is not homologous to this metazoan β-galactosidase, similar sequences to the *B. plicatilis* β-galactosidase sequence were identified in UniProtKB using BLASTX, and a maximum likelihood phylogeny was constructed using these hits and the WAG+I+G model of protein evolution. This clearly shows *B. plicatilis* β-galactosidase to be metazoan in origin (fig. 2.1), grouping monophyletically with metazoan sequences (aLRT=0.98).

Figure 2.1. Maximum likelihood gene tree of the translated amino acid sequence of metazoan β-galactosidase in *B. plicatilis* and other samples, WAG+I+G model of protein evolution. Support values (aLRT) above 0.5 displayed. Species coloured by kingdom (blue = bacteria, pink = plant, red = metazoa).

***
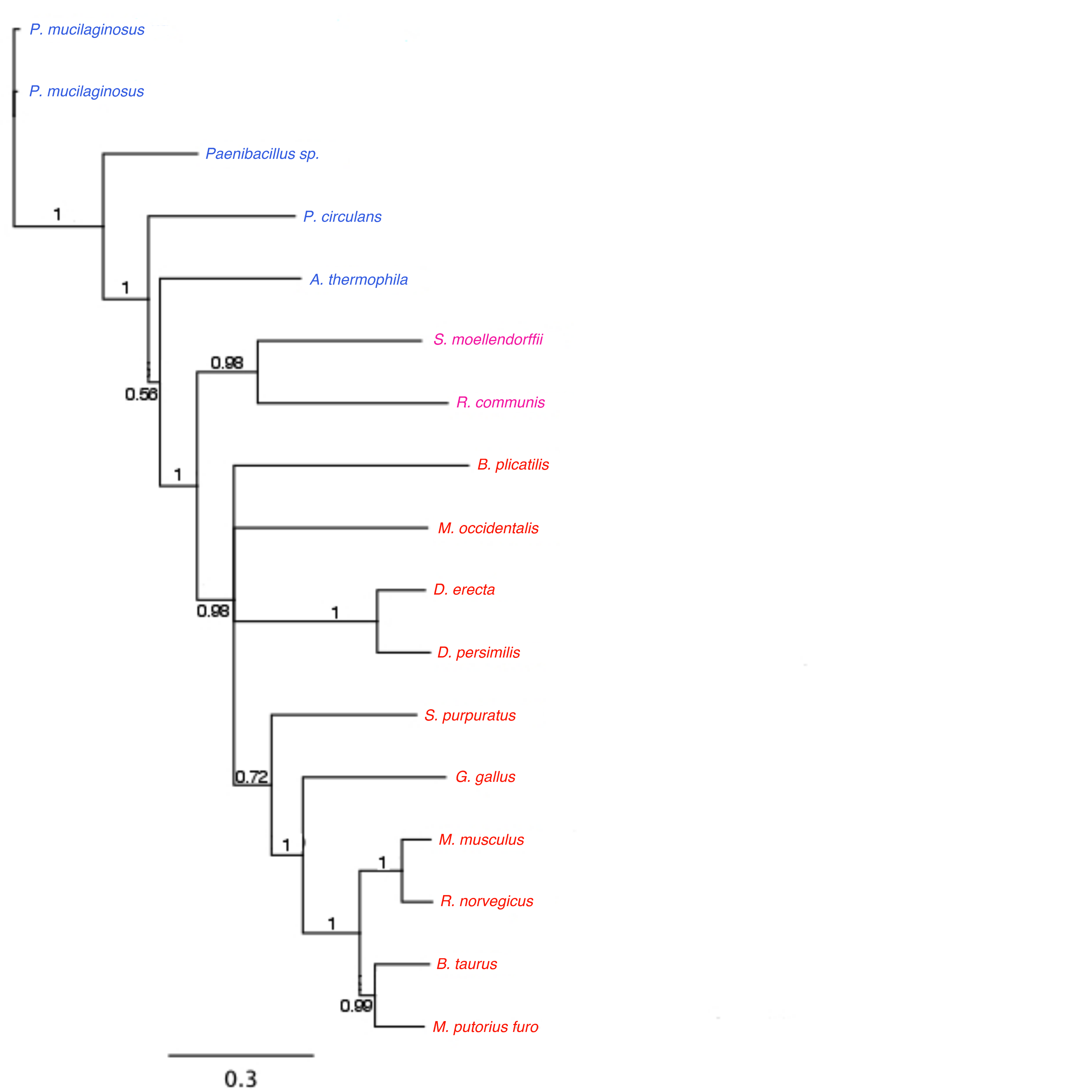
***

Based on the identification of AV10092, AV10109 and AV10027 in both Adineta and Rotaria transcriptomes, we predicted that these genes were acquired before the divergence of the families Adinetidae and Philodinidae. Consistent with this prediction these genes were successfully amplified from the gDNA of species from both families (fig. 2.2). AV10092 was also amplified in Habrotrocha ligula (family Habrotrochidae), pointing to an even more ancient origin of this gene. AV10134 was only identified in the A. ricciae transcriptome, and so was predicted to have been acquired by Adinetidae after the divergence of this lineage. This prediction was borne out by the failure to amplify AV10134 from gDNA of species outside of Adinetidae (fig. 2.2).

Figure 2.2. presence of candidate genes AV10027, AV10092, AV10109 and AV10134 determined by (a) presence of PCR product only (blue) or (b) successfully sequenced PCR product (green).

| **Family** | **Species** | **AV10027** | **AV10092** | **AV10109** | **AV10134** |
| --- | --- | --- | --- | --- | --- |
| Adinetidae | *Adineta barbata* |  |  |  |  |
|  | *Adineta cuneata* |  |  |  |  |
|  | *Adineta gracilis* |  |  |  |  |
|  | *Adineta ricciae* |  |  |  |  |
|  | *Adineta steineri* |  |  |  |  |
|  | *Adineta tuberculosa* |  |  |  |  |
|  | *Adineta vaga* |  |  |  |  |
|  |  |  |  |  |  |
| Philodinidae | *Dissotrocha aculeata* |  |  |  |  |
|  | *Philodina megalatrocha* |  |  |  |  |
|  | *Philodina citrina* |  |  |  |  |
|  | *Rotaria mento* |  |  |  |  |
|  | *Rotaria rotatoria* |  |  |  |  |
|  | *Rotaria magnacalcarata* |  |  |  |  |
|  | *Rotaria socialis* |  |  |  |  |
|  | *Rotaria sordida* |  |  |  |  |
|  | *Rotaria tardigrada* |  |  |  |  |
|  |  |  |  |  |  |
| Habrotrochidae | *Habrotrocha ligula* |  |  |  |  |

In further support of predictions of HGT events illustrated in fig 2.2, phylogenetic analyses (fig 2.3-2.6) demonstrate that each of the four foreign genes examined here are monophyletic in bdelloid species, and therefore arose from a single uptake event, not from multiple independent origins.

Figure 2.3. Maximum likelihood gene tree of the translated amino acid sequence of AV10092 in bdelloid samples and out-groups, WAG+I+G model of protein evolution. Support values (aLRT) above 0.5 displayed. Species coloured by kingdom (blue = bacteria, pink = plant, orange = metazoa, purple = fungi, red = bdelloid).


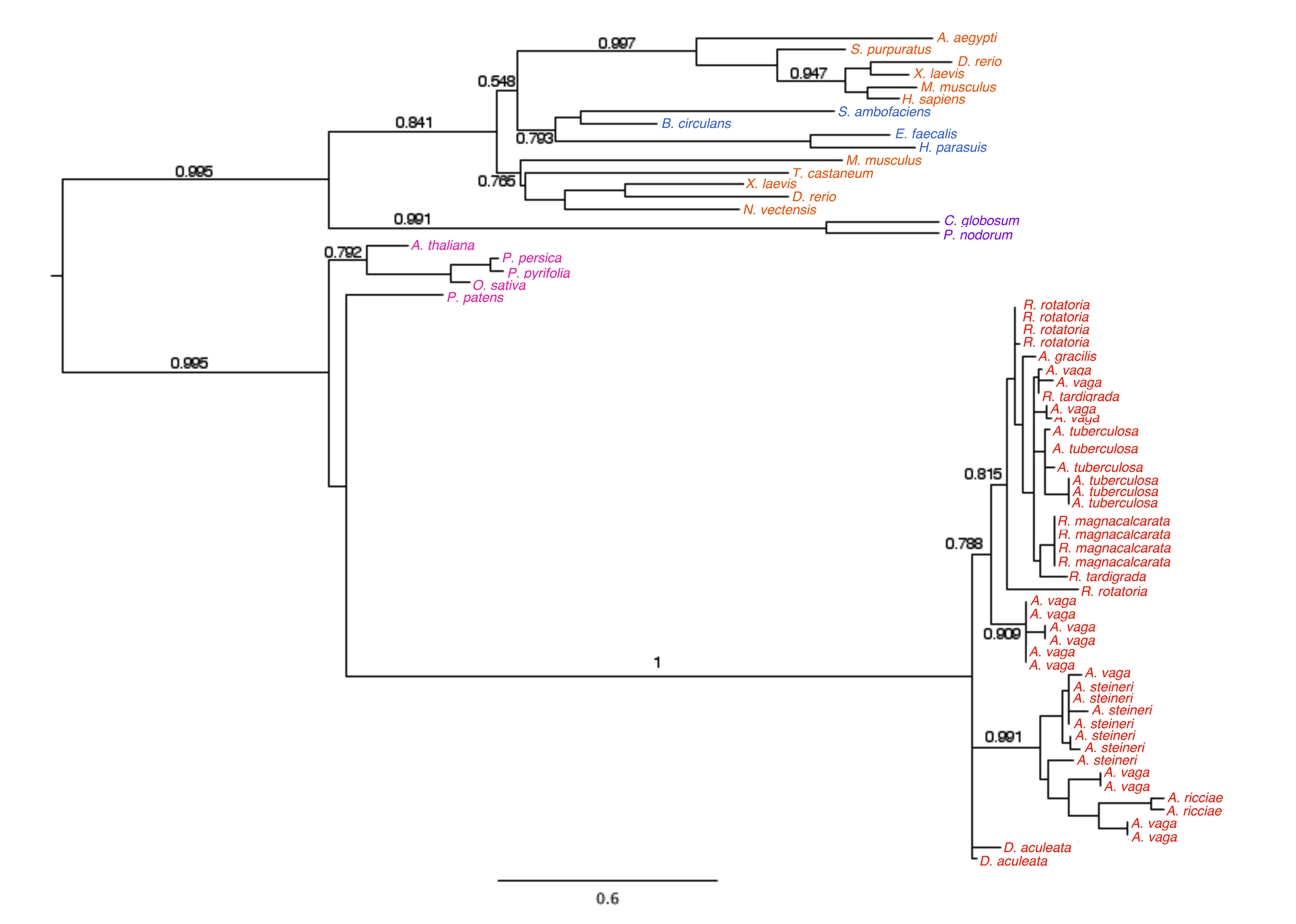


Figure 2.4. Maximum likelihood gene tree of the nucleotide sequence of AV10027 in bdelloid samples and outgroups, K80+G model of nucleotide substitution. Support values (aLRT) above 0.5 displayed. Species coloured by kingdom (blue = bacteria, red = metazoa).

**
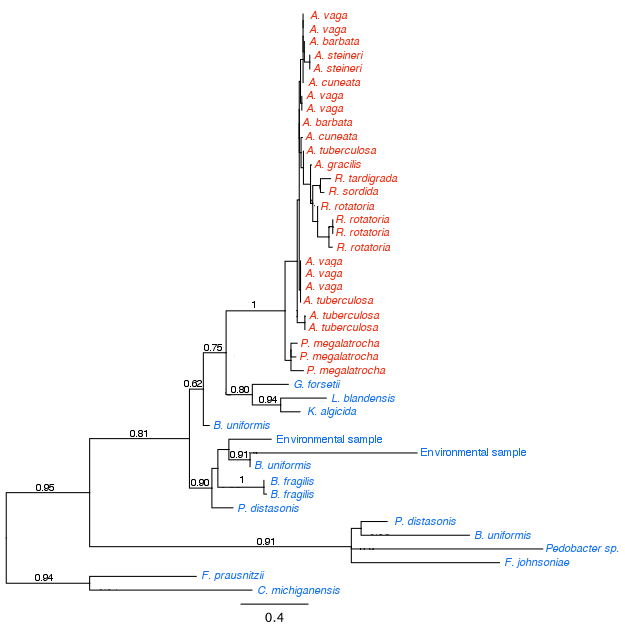
**

Figure 2.5. Maximum likelihood gene tree of the translated amino acid sequence of AV10109 in bdelloid samples and outgroups, LG+I+G model of protein evolution. Support values (aLRT) above 0.5 displayed. Species coloured by kingdom (blue = bacteria, pink = plant, orange = metazoa, purple = fungi, yellow = protista, red = bdelloid).

Figure 2.6. Maximum likelihood gene tree of the translated amino acid sequence of AV10134 in bdelloid samples and outgroups, WAG+I+G model of protein evolution. Support values (aLRT) above 0.5 displayed. Species coloured by kingdom (blue = bacteria, pink = plant, orange = metazoa, purple = fungi, grey = archaea, red = bdelloid).


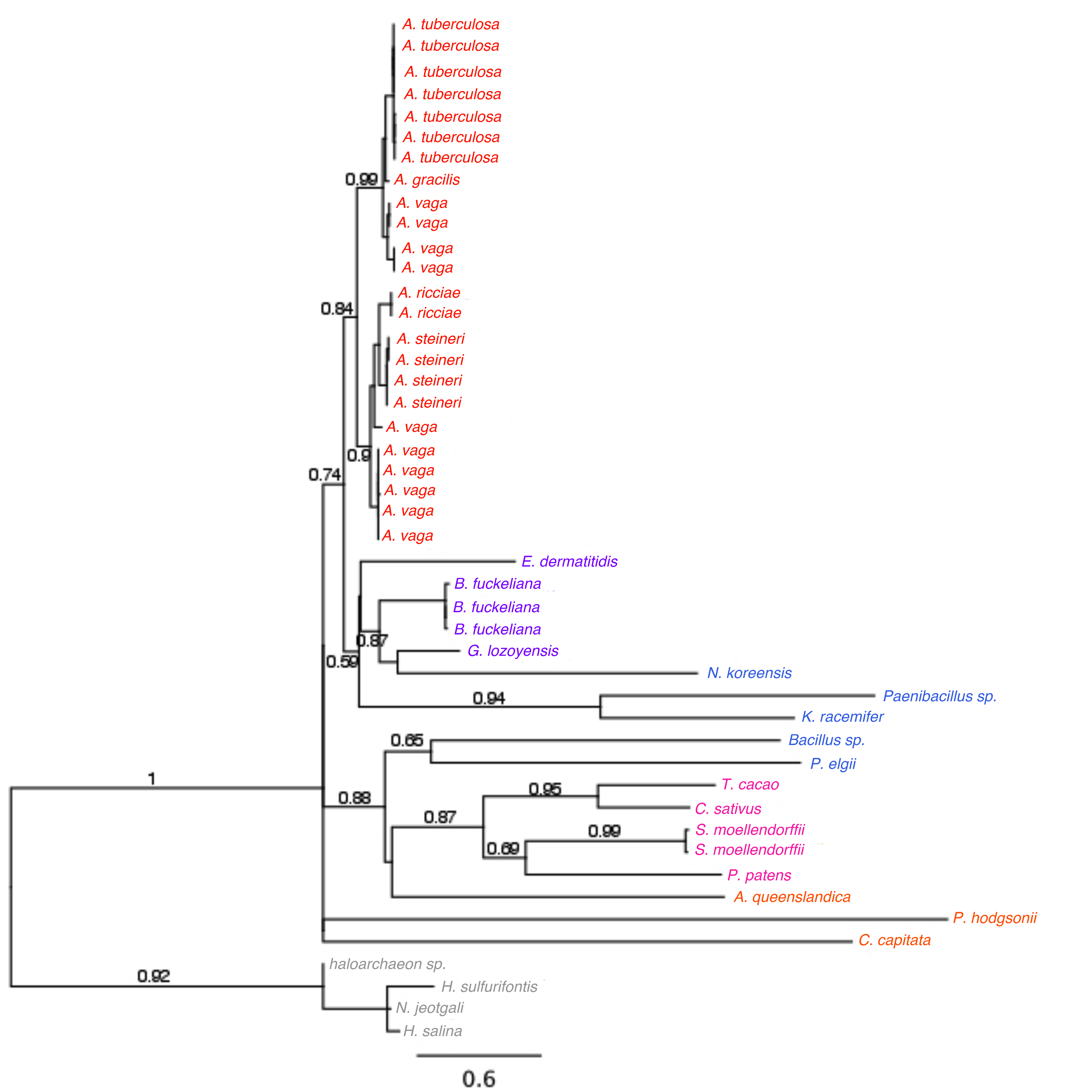

Supplement: Additional file 2: — Survey of four foreign genes in the gDNA of a selection of bdelloid species. (DOCX 4251 kb) [file 12915_2015_202_MOESM2_ESM.docx]
