# Supplementary material for: Horizontal gene transfer in bdelloid rotifers is ancient, ongoing and more frequent in species from desiccating habitats
Source: BMC Biol. 2015 Nov 4;13:90. doi: 10.1186/s12915-015-0202-9 (PMC4632278; doi:10.1186/s12915-015-0202-9)
Supplement: Additional file 3: — Comparison of divergence and selection in native and foreign genes. (DOCX 93 kb) [file 12915_2015_202_MOESM3_ESM.docx]

*Additional File 3 – Comparison of divergence and selection in native and foreign orthologs*

**Methods**

For the same orthologous gene trees the sum of branch lengths within each tree was calculated using (sum(phylogeny$edge.length)) in the APE package in R [1] as a measure of the variability of the gene. dN/dS ratio was calculated across *Rotaria* orthologs in PAML [60]. Positive selection among orthologs was detected using the likelihood ratio test between the codon-substitution models M1a (nearly neutral; ω ≤ 1) and M2a (positive selection; including a category of sites with  > 1) in PAML [2].

**Results**

There was no significant difference in the rate of divergence between native and foreign transcripts in *Rotaria* species (mean metazoan substitutions per site = 0.370±0.04, mean foreign substitutions per site = 0.349±0.01, t-test t=0.431, df=3327.0, p = 0.67) in the set of 3330 transcripts common to all five species in reciprocal BLAST matches. To test for evidence of positive selection on genes, likelihood ratio tests were used to compare the codon-substitution models M1a (nearly neutral; a proportion p1 of sites have ω < 1 and p2 sites have ω = 1) and M2a (positive selection; includes an additional proportion p3 of sites with ω > 1) in the same set of orthologous genes using PAML. Most genes were under purifying selection and the M1a model could not be rejected: M2a was only a significantly better fit in 5/197 (2.5%) of foreign orthologs and 65/3133 (2.1%) of metazoan orthologs. The dN/dS ratio was not significantly different between metazoan (mean = 0.136±0.003) and foreign orthologs (mean = 0.114±0.005, t-test, t = -0.64, df = 3184.7, p-value = 0.52), although median dN/dS was higher in foreign orthologs (0.101) in comparison to metazoan sequences (0.084, Mood’s median test, Z = -3.07, p = 0.002).

References

1. Paradis E, Claude J, Strimmer K. **APE: Analyses of Phylogenetics and Evolution in R language**. *Bioinformatics* 2004, **20**:289–290.

2. Yang Z. PAML 4: **Phylogenetic Analysis by Maximum Likelihood**. *Mol Biol Evol* 2007, **24**:1586–1591.
